# Advances in Assistive Electronic Device Solutions for Urology

**DOI:** 10.3390/mi13040551

**Published:** 2022-03-30

**Authors:** Kieran Holmes-Martin, Minghui Zhu, Shujun Xiao, Faezeh Arab Hassani

**Affiliations:** Department of Electrical and Electronic Engineering, University of Bristol, 75 Woodland Road, Bristol BS81UB, UK; fs20507@bristol.ac.uk (K.H.-M.); oi20904@bristol.ac.uk (M.Z.); nt20838@bristol.ac.uk (S.X.)

**Keywords:** urology, electronic device solutions, robotic surgery, bladder monitoring and stimulation, bladder and sphincter prosthesis, artificial bladder, artificial sphincter, overactive bladder, underactive bladder, other-urinary-affecting disorders

## Abstract

Recent technology advances have led urology to become one of the leading specialities to utilise novel electronic systems to manage urological ailments. Contemporary bladder management strategies such as urinary catheters can provide a solution but leave the user mentally and physically debilitated. The unique properties of modern electronic devices, i.e., flexibility, stretchability, and biocompatibility, have allowed a plethora of new technologies to emerge. Many novel electronic device solutions in urology have been developed for treating impaired bladder disorders. These disorders include overactive bladder (OAB), underactive bladder (UAB) and other-urinary-affecting disorders (OUAD). This paper reviews common causes and conservative treatment strategies for OAB, UAB and OUAD, discussing the challenges and drawbacks of such treatments. Subsequently, this paper gives insight into clinically approved and research-based electronic advances in urology. Advances in this area cover bladder-stimulation and -monitoring devices, robot-assistive surgery, and bladder and sphincter prosthesis. This study aims to introduce the latest advances in electronic solutions for urology, comparing their advantages and disadvantages, and concluding with open problems for future urological device solutions.

## 1. Introduction

Urology is a diverse subbranch of medicine that amalgamates the management of medical and surgical conditions of the male and female urinary tract and the male reproductive organs [1,2]. The most common medical conditions in urology are urinary tract infection (UTI), benign prostatic hyperplasia (BHP), and urinary incontinence [3,4,5,6]. Common surgical conditions that can be treated by surgery are bladder or prostate cancer, kidney and ureteral stones, traumatic injury, interstitial cystitis and correcting stress incontinence [7,8,9,10]. Pharmacotherapy is often suggested for treating medical conditions, whereas surgical conditions require surgeon expertise [11].

Urological disorders influence the lives of millions of people worldwide each year. In the UK alone, 10,000 people are diagnosed with cancer yearly, making it the 11th most common cancer [12]. An estimated 3–6 million people in the UK (i.e., 5% men and 13% women) have urinary incontinence [13], interstitial cystitis affects 400,000 people (90% of whom are women) [14], and 1 in 2000 men and between 50% and 60% of women will develop a UTI at least once in their lifetime [15]. A few research groups have studied racial and ethnic differences in the prevalence of urological disorders. Zuo et al. [16] conducted a study to identify differences in severity of overactive bladder (OAB) in women of different races, and Van Den Eeden et al. [17] sought to examine if there are racial or ethnic differences in lower urinary tract symptoms (LUTS) in men. A recent study showed more than 70% of urinary incontinence is associated with bother regardless of race or ethnicity [18].

Many urological disorders are treated using conservative management methods such as drug and pharmacotherapy [19,20,21], physiotherapy [22], pelvic floor exercises [23,24,25,26], hormonal therapy [27], radiotherapy [28], lifestyle changes [21,29], and intermittent and indwelling urinary catheters [20,30,31,32]. However, many of these methods often fail and impose a detrimental effect on patients’ quality of life. Fortunately, with the advancement of new technology such as soft flexible electronics and biocompatible materials, in the recent decades, urology has become one of the leading specialities to utilise novel surgical and electronic technologies [33].

**Figure 1:**
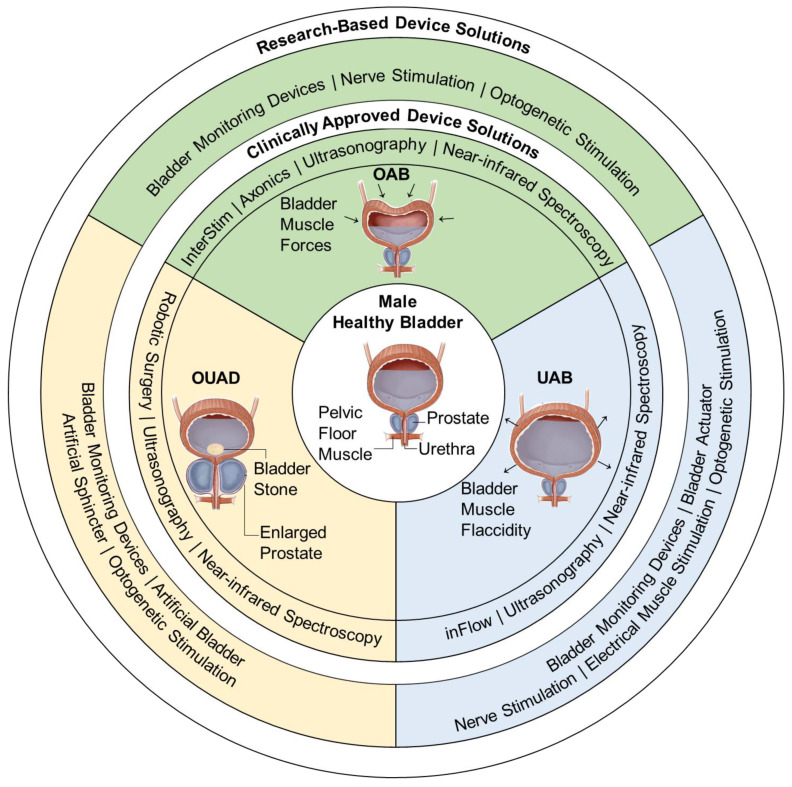
Clinically approved and research-based electronic device solutions for overactive bladder (OAB), underactive bladder (UAB) and other-urinary-affecting disorders (OUAD). Clinically approved electronic solutions include InterStim [40], Axonics modulation technologies [53], inFlow [39], robotic surgery [54,55], ultrasonography systems [56,57] and near-infrared spectroscopy [58]. Research-based device solutions include bladder-monitoring devices [59,60,61,62,63,64], nerve stimulation [46], optogenetic stimulation [43,44,45], electrical muscle stimulation [65,66], bladder actuators [47,48,49], artificial sphincter [67,68], and artificial bladder [50,51,52].

Figure 1 subdivides urological disorders into OAB, underactive bladder (UAB) and other-urinary-affecting disorders (OUAD) and presents clinically approved and novel electronic assistive solutions. Robot-assistive surgery [34,35,36,37,38], and invasive (e.g., InterStim) and non-invasive devices (e.g., inFlow) [19,39,40] are presented as current clinically approved treatments. However, due to the many adverse side-effects, assistive electronic devices better suited to manage urological conditions are being explored. Several reviews have investigated the bladder-monitoring systems [41,42]. However, the recent development of flexible electronics and biocompatible materials provides a range of novel electronic solutions such as bladder-stimulation devices [43,44,45,46], bladder actuators [47,48,49] and artificial prosthetic devices [50,51,52] that can assist in emptying the bladder. Therefore, a gap in reporting new technologies for urology was observed. The purpose of this study is to provide a broad perspective to all urological monitoring and assistive electronic solutions for the first time.

**Figure 2:**
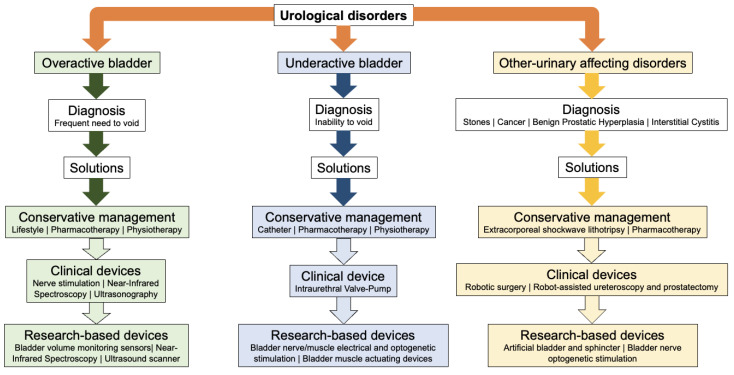
A summary of steps that are considered for each urological disorder in this study.

This paper is organised into the following sections. Section two describes the methodology for conducting the review. Urological disorders are divided into OAB, UAB, and OUAD and presented in order in sections three, four, and five. Each section describes the causes and complications caused by the disorder, current conservative and clinically approved management strategies and state-of-the-art research-based electronic solutions. Figure 2 summarises the steps of this study for each disorder. A systematic review of materials, fabrication methods and potential challenges for each research-based solution is provided for each urological disorder. Section six provides a summary and future directions for electronic solutions in urology.

## 2. Methodology

Well-known databases and digital libraries such as PubMed, PubMed Central (PMC), IEEE, National Center for Biotechnology Information (NCBI) and Nature were used to search for relevant materials for this study. Due to the broad range of conditions related to urology, several keyword combinations were used with “OAB” and “UAB”. Such keywords included “bladder monitoring”, “electronic devices”, “bladder stimulation”, “bladder prosthesis”, “artificial bladder/sphincter”, and “electronic and device solutions”. Since OUAD is a new term for defining other urological disorders unrelated to the bladder, keywords such as “renal”, “kidney”, and “prostate” were used. Primary articles related to electronic device solutions in urology are summarised in Table 1. After investigation, a total of 62 papers were selected for the review. Papers with detailed engineering descriptions on electronic advancements related to urology were selected. Papers that lacked a detailed description of the device solution were disregarded.

## 3. Overactive Bladder

### 3.1. Diagnosis

OAB is a chronic medical condition and is characterised by the International Continence Society (ICS) as ”urgency, with or without urge incontinence, usually with increased daytime frequency and nocturia”, and can have a substantial impact on quality of life in both men and women [69,70]. OAB can be a demeaning condition that disrupts myriad activities of daily living—people suffering from OAB are likely to limit participating in social events, partaking in physical activities and can disrupt work performance by decreased productivity due to frequent trips to the toilet [71]. Most people with OAB experience frequent, sudden urges to urinate, often accompanied by leaking urine—with nocturia being the most bothersome symptom [6].

Men, women, and children across all ages can experience symptoms of OAB—but the prevalence is concentrated in older women, generally in patients over 40 years old [72]. A large study conducted by the National Health Service (NHS) in the UK discovered that approximately 1 in 6 women experience OAB symptoms to some degree [73].

The causes of OAB are multifaceted and are not completely understood. However, a few theories have been proposed to explain the pathophysiology of OAB—presented in Table 2. OAB is often observed in many neurological failures and myogenic conditions. Neurogenic OAB appears to stem from either abnormal central processing of afferent input, increased afferent input from the bladder or physical damage to the spinal cord [74], whereas myogenic OAB stems from involuntary detrusor contraction, resulting in an increase in random activity [74].

### 3.2. Clinically Approved Management Strategies

#### 3.2.1. Conservative Management Strategies

Many causes of OAB are unforeseeable, implying there is no exact way to prevent OAB. However, the most common treatment for OAB is conservative management—this involves medication, various lifestyle interventions or physiotherapy [29], as displayed in Table 3. Lifestyle changes are based on expert opinion but have limited scientific evidence; however, a healthier lifestyle can have minimal risk and often improves overall health and well-being. Common lifestyle changes include diet management achieved by altering fluid intake, limiting the intake of caffeine and alcohol, and utilising the aid of a nutritionist [5,21,22,29]. If symptoms persist, physiotherapy strategies such as bladder and pelvic floor muscle training can help manage OAB. These physiotherapy strategies aim to increase bladder capacity and reduce detrusor contractions, leading to reduced urge incontinence [80]. The use of pharmacological treatments can benefit people with OAB if physiotherapy is unsuccessful. The gold-standard pharmacological treatment method is the use of antimuscarinic (also known as anticholinergic) drugs to relax the detrusor muscle and improve patients’ symptoms [5]. The drugs work by producing a competitive inhibitor of the cholinergic-muscarinic receptor, resulting in fewer detrusor contractions [5]. There are other drug treatments, shown in Table 3. Although antimuscarinic drugs can improve symptoms, there can be many adverse side effects—the most common ones include dry mouth and eyes, constipation, and heart palpitations [21]. There have also been reports of cognitive side-effects involving changes in memory, blurred vision, somnolence, hallucinations, confusion, and delirium, which all become more prevalent as age increases [81].

#### 3.2.2. Nerve Stimulation Devices

Studies have shown that within a year, between 30% and 70% of patients with OAB discontinue pharmacotherapy [82,83]. Hence, various stimulation devices are an alternative next-in-line treatment option for OAB and although they are more invasive, they have shown better improvement for OAB [53].

##### InterStim

**Figure 3:**
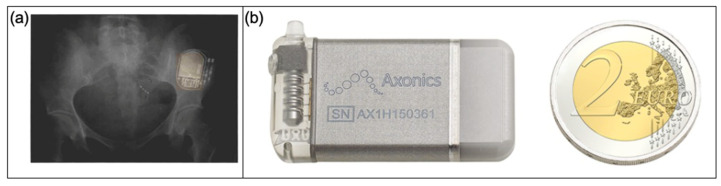
Nerve stimulation devices. (**a**) An anterior–posterior radiograph showing the InterStim device in upper buttock, with a lead electrode in sacral vertebrae (S4) foramen [40]; (**b**) the Axonics neurostimulator device next to a coin [84]. Copyright 2018, John Wiley and Sons.

Patients may opt for implantation of the InterStim device, manufactured by Medtronic, Inc [40]. The device has a battery life in the range of 6–10 years, depending on the setting. The device is implanted in the upper buttock region and has connecting leads to the sacral nerve. The stimulation of the sacral nerve can lead to inhibitory reflex, resulting in the cessation of involuntary bladder voiding. All controls on the neurostimulator are accessible using an external programmer, and the patient can control the settings on the stimulation device to adjust for comfort level. Figure 3a presents a radiograph showing the neurostimulator device in the upper buttock [40]. This method requires invasive surgery and manipulation of the nerves which has limited its use.

##### Axonics Modulation Technologies

Another sacral nerve stimulation device to combat OAB has been developed by Axonics modulation technologies [84]. The device is 60% smaller than the current InterStim device at 5 cm^3^ in volume and is shown in Figure 3b. The device is a rechargeable neurostimulator connected to an impulse generator that is implanted in the upper buttock and has a tined lead with four electrodes percutaneously inserted through the sacrum. Furthermore, the device has a 15 year battery life and can alter output voltage based on tissue impedance. Additionally, the device is currently approved for use in a magnetic resonance imaging (MRI) scanner up to 1.5–3 Tesla [53].

#### 3.2.3. Ultrasonography Systems

The following non-invasive bladder-monitoring methods can be applied to OAB, UAB and OUAD. Ultrasonography primarily use signals or images to assess the volume of the bladder without the need to injure the patient through invasive operations [42]. Most clinical devices are operated based on ultrasonography techniques and are divided into stationary and portable devices. [56]

An example of common stationary ultrasonography is the Vingmed CF 800ä [56], which is a multi-specialty ultrasound system providing high-resolution digital scanning, whereas the BladderScanâ BVI 6400ä [57] is a dedicated portable ultrasound device that uses the three-dimensional (3D) mode of ultrasound imaging technology to provide an image of the bladder and abdominal space. The BladderScan is a portable 1.85 Kg battery-powered ultrasonic instrument with a digital display screen and a handheld scanning head. In a study conducted by Huang et al. [57], it was discovered that the stationary ultrasonography had a significantly lower absolute error determining the volume of the bladder than the handheld device. However, since the stationary device is more complex, it requires more time to operate, making the portable BladderScan more appealing.

#### 3.2.4. Near-Infrared Spectroscopy Systems

Near-infrared spectroscopy (NIRS) is a non-invasive technology that utilises energy from light in the near-infrared spectrum to monitor changes in local blood flow and haemodynamics and detect differences in tissue oxygen delivery, consumption and utilisation [58]. NIRS is particularly relevant to the study of the bladder due to the negative impact of haemodynamic muscle detrusor disorders and oxygenation of normal voiding function [85]. NIRS can be used to monitor bladder fullness due to the absorption of light in particular wavelengths. Water in urine has an absorption peak at 975 nm—this peak can be used to determine the volume of urine in the bladder to distinguish between a full and an empty bladder [85].

### 3.3. Research-Based Solutions

#### 3.3.1. Bladder-Monitoring Devices

For many urological conditions under OAB, UAB and OUAD, the monitoring of renal function can be vital for patients in acute care settings. One indicator for healthy kidneys is bladder function and volume. Currently, a urinary catheter with an integrated pressure sensor is the most common method used to monitor bladder performance; however, this induces a great risk of contracting a UTI [86]. To mitigate this issue, there has been development in electronic monitoring methods that combine with state-of-the-art implantable and wearable devices to observe bladder function.

##### Near-Infrared Spectroscopy Systems

**Figure 4:**
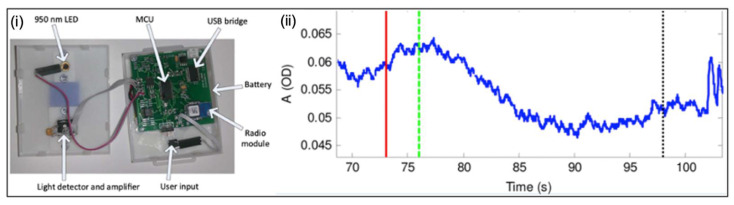
Near-infrared spectroscopy method. (**i**) NIRS device used for monitoring urine in the bladder; (**ii**) attenuation detection during voiding. The red, green and black lines are permission to void, beginning and end of voiding, respectively [87]. Copyright 2014, IEEE.

Molavi et al. [87] have presented a wireless NIRS device (Figure 4) that consists of a 960 nm light-emitting diode (LED) as a source LED, and a detector approximately 3 cm away from the LED source. Under operation, the source LED emits light, and the detector receives the reflection. The absorption of emitted light by chromophores in the tissue attenuates the light; then, using machine learning algorithms, the attenuation can be used to determine the fullness of the bladder. Furthermore, NIRS can be applied to many other urological conditions besides bladder monitoring—cryptorchidism, testicular torsion, erectile dysfunction, renal oxygenation, and muscle metabolism in renal dysfunction are among a few [88].

##### Ultrasound Scanner Systems

**Figure 5:**
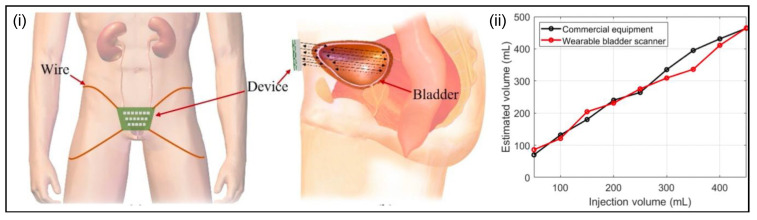
The proposed wearable bladder scanner system. (**i**) The front and side view of the device; (**ii**) estimated bladder volume measured by the proposed device and commercial equipment for various injection volumes [89].

Jo et al. [89] have developed a wearable bladder scanner system that can continuously measure bladder volume in daily life. The system (Figure 5) consists of a two-dimensional ultrasound transducer 5 × 5 array, which includes integrated forward-looking piezoelectric transducers with thin substrates. The device is placed 2 cm above the pubic bone and is secured with wires or belts. The sensing system uses low-profile ultrasound transducers with a forward-looking pulse-echo technique to estimate urinary bladder volume. The volume is estimated by interpolating the shape of the bladder based on measurements of the location of the bladder wall. The study showed that estimations of the bladder volumes with various injection volumes obtained an average absolute error of 24 mL compared to commercial equipment at 29 mL (Figure 5). The experiment demonstrated the feasibility of two-dimensional ultrasound transducer array systems. The proposed method for estimating bladder volume has the advantage of being faster than contemporary methods but have the drawback of being cost-ineffective due to their scanning shape.

##### Bioimpedance Sensors

**Figure 6:**
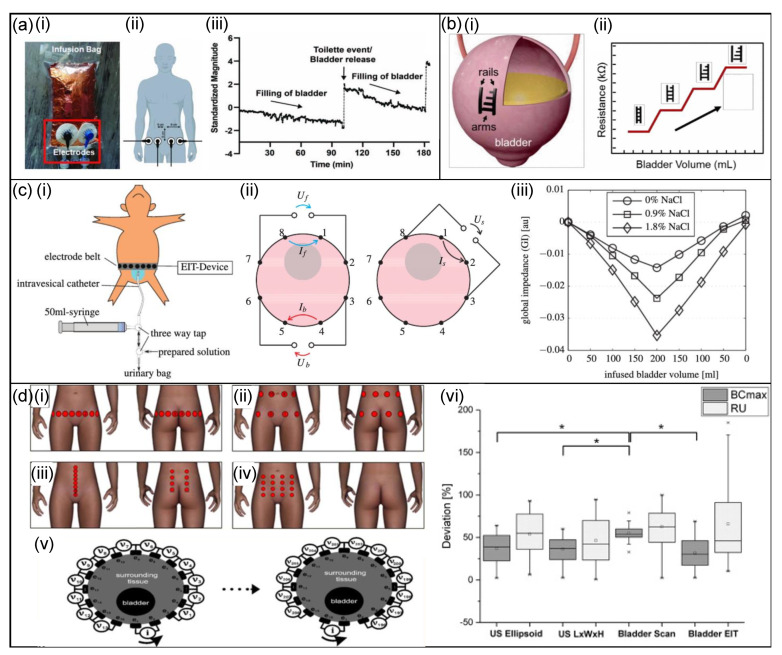
Bladder-monitoring devices. (**a**,**i**) The bioimpedance sensor on an infusion bag, (**ii**) a schematic of the position of electrode position on the body, and (**iii**) impedance versus time characteristics [59]. Copyright 2020, IEEE. (**b**,**i**) The multi-level resistor bioimpedance sensor, and (**ii**) the discrete changes in the resistance of the sensor due to the number of attached arms [61]. Copyright 2017, IEEE. (**c**,**i**) The experimental setup of in vivo measurements showing the electrode belt with attached EIT device, (**ii**) the impedance ratio method using three tetrapolar measurements, and (**iii**) the influence of urine conductivity on EIT global impedance [91]. (**d**,**i**–**iv**) Four different electrode configurations for EIT measurements—each configuration has 16 electrodes, (**v**) global impedance method displaying the 16 electrode ring arrangement, and (**vi**) the deviation of different measurement tools for cystovolumetry compared to the actual bladder volume [92].

A non-invasive wearable bioimpedance system to wirelessly monitor bladder filling has been proposed by Reichmuth et al. [59]. Sensing the electrical impedance of the bladder during filling via electrodes attached to the skin allows for long-term monitoring without the need for invasive operations. The wireless sensor system incorporates a micro-controller and Bluetooth, power management and several sensors such as electrocardiography (ECG) electrodes, a temperature sensor, an inertial measurement unit (IMU) and a bioelectrical impedance analysis (BIA) unit. Data are processed directly onboard with a Bluetooth (BTLE) 5.0 wireless interface using a 64 MHz ARM Cortex-M4F micro-controller with a 32 bit processor (the nRF52832 by Nordic Semiconductor [90]). All components are powered by a Li-ion battery and the voltage is regulated to a constant 3.3 V level. Figure 6a presents the experimental setup on the infusion bag, and the intended position on a human body, respectively. Figure 6a also presents bioimpedance versus time characteristics and shows that changes in bioimpedance can accurately measure the volume of the bladder. The system exhibited low power consumption of 80 µW for one acquisition every 5 min, providing a long-term wearable monitoring system.

Another implantable bladder volume-measuring device based on bioimpedance is proposed by Kim et al. [61] and is shown in Figure 6b. The sensor is composed of biocompatible polypyrrole/agarose hydrogel composite organised in a multi-level resistor ladder—this design allows the sensor output to be resilient to long-term drift, hysteresis, and degradation of the sensor material. The ladder legs are 40 × 5 × 2 mm (width × length × height). The system attaches to the outside of the bladder and when the volume changes, the number of arms that are in contact with the opposite rail changes, resulting in a discrete change in the total resistance of the sensor; the overall impedance is calculated based on the number of arms that are attached to both legs. Through ex vivo experiments on extracted pig bladder, the sensor was successfully demonstrated to alter resistance as the bladder’s volume increased (Figure 6b). The measurement results show irregular changes in resistance due to insecure attachment to the bladder.

Further work in this field has been conducted by Schlebusch et al. [91]. They have presented an extension of the extracorporeal bioimpedance approach to multi-electrode electrical impedance tomography measurement (EIT). The system is an elastic electrode belt with 16 conductive silicone electrodes placed towards the lower torse with an experimental EIT system attached (Figure 6c). The electrode belt is placed as caudal as possible while maintaining stable electrode contact and takes three-dimensional measurements to help reduce distorting influences and provide more accurate measurements over tetrapolar measurements. The device uses adjacent injection and measurement patterns, resulting in a total of 208 readings per measurement and these measurements are compared to a reference measurement of an empty bladder to reconstruct an image. During in vivo experiments, it was shown that liquids of different conductivity had a significant effect on global impedance. To mitigate this influence on volume estimation, the impedance ratio method was used. The idea is to correlate volume with a ratio of three different impedance values (three measurements: front, back and side) at the bladder level around the abdomen to reduce the influence of unknown urine conductivity on the volume estimate.

Leonhauser et al. [92] conducted a study to compare the measurement accuracy of EIT to the accuracy of ultrasound (US) and bladder scan (BS) volumetry in healthy volunteers. For the EIT of the bladder, a commercial device (Goe MF II) was used with four different configurations of 16 standard ECG electrodes attached to the lower abdomen (Figure 6d). It was shown that EIT has considerable potential as a cystovolumetry system in comparison to standard ultrasound-based measurements but requires the optimisation of electrode configuration and calibration and minimising the movement artefacts.

##### Pressure Sensors

**Figure 7:**
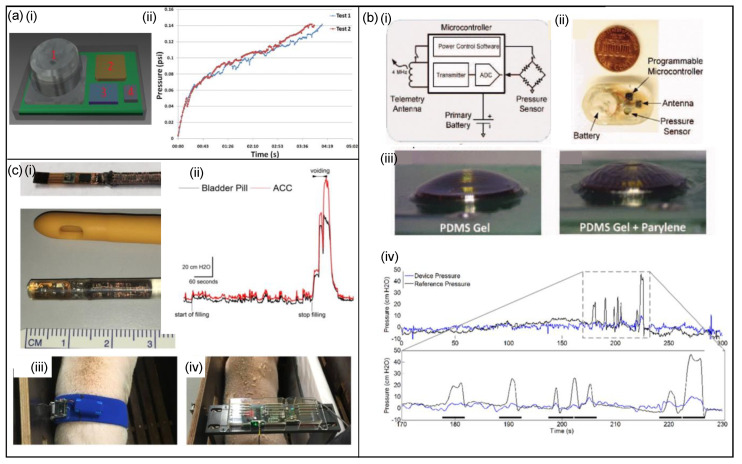
Bladder−monitoring devices. (**a**,**i**) The schematic of the pressure sensor CAD design. Component 1 is an MS5534C miniature pressure sensor, component 2 is the ATtiny43u microcontroller, component 3 is the ZL70101 transmitter, and component 4 is a standard oscillator to be used for the miniature pressure sensor; (**ii**) a pressure−time curve [62]. Copyright 2010, IEEE. (**b**,**i**) The wireless pressure sensor circuit diagram, (**ii**) the size compared to a coin, (**iii**) the different polymer coatings, and (**iv**) graphs of catheter and wireless pressure monitoring pressure recordings versus time [93]. Copyright 2016, IEEE. (**c**,**i**) The bladder pill device, the non−encapsulated and the encapsulated bladder bill, (**ii**) details of a single void marked on a graph, (**iii**) the waistband device, and (**iv**) rectangular frame setup [94].

Weaver et al. [62] have presented a pressure-sensing system that is attached to the inside of the bladder wall to collect and wirelessly transmit bladder pressure data over a 12–24 h period. The system attaches to the inside of the bladder wall and has been designed to be fabricated using low-power and low-current components and has the dimensions 11.5 × 17 × 8 mm (length × width × thickness). The design is displayed in Figure 7a and consists of a piezoresistive pressure sensor (capable of pressure measurements up to 15.9 psi absolute), a microcontroller, a wireless transmitter, and a battery that is contained in a layer of polydimethylsiloxane (PDMS) to seal the electronic components from the bladder environment. A pressure–time curve is presented in Figure 6a showing an initial large slope for the first few millilitres due to a column of hydrostatic pressure. The device is intended to be implanted using a cystoscope and data will be transmitted to a tiny receiver attached to a belt worn outside the body. The user would conduct their normal daily routine for 12–24 h as the data accumulate—after which, the patient shall return to the doctor’s office for the data to be downloaded and analysed.

Moreover, Majerus et al. [93] have designed an implantable wireless and real-time bladder pressure-monitoring system and provided a basis for closed-loop neuromodulation in patients with lower urinary tract dysfunction. The packaged sensor and testing results are shown in Figure 7b. Figure 7b shows the new bladder pressure-monitoring system—it includes an ultra-low-power application-specific integrated circuit (ASIC), a micro-electro-mechanical (MEMS) pressure sensor, radio frequency (RF) antennas, and a rechargeable battery, which forms a complete implantable microsystem. The system is powered by a wireless power transmitter, while the user sleeps on a cushion with an embedded RF induction coil. Continuous real-time bladder pressure can be measured by the wireless pressure sensor within the bladder wall, and then the pressure data can be transmitted to an external receiver via a telemetry antenna. With a non-hermetic polymer packaging process, the pressure sensor was covered by a convex drop of PDMS gel (Figure 7b), and the pressure diaphragm was fixed to the inner mould wall by PDMS before the chronic in vivo sensor packing test. Then, verified by in vivo wireless bladder pressure sensing, the data quality was sufficient to detect bladder compressions in large animals, with an average correlation coefficient of 0.90, which correlates well with the reference catheter device and the trend of the pressure curves is consistent (Figure 7b).

Furthermore, Soebadi et al. [94] have created a wireless intravesical device for real-time bladder pressure measurement in awake pigs. The measurement system consists of the bladder pill and a corresponding external device (Figure 7c). The pill contains an MS5637 pressure sensor microchip and a custom three-dimensional power-receiving coil, all encapsulated in medical-grade silicone tubing at approximately 4.6 mm in diameter. The pill is designed for soft encapsulation and flexibility for easy urethral insertion in humans. The external device consists of a transmitting coil to energise and exchange data in addition to the needed power and communication circuits. There were two different designs for the external device: a waistband and a free-standing rectangular frame (Figure 7c). The waistband was used for in vitro bench studies and had a sinusoidal coil layout and covered 3 mm-thick neoprene. The frame was designed to avoid direct cutaneous contact and was fabricated from a 15 mm-thick polyvinyl chloride (PVC) sheet which encases a single-turn inductive loop. In experiments in awake pigs, the bladder pill showed strongly correlated measurements compared with three-way air-charged catheters (ACC) connected to a medical urodynamic system, as shown in Figure 7c.

Basu et al. [95] have developed a wireless pressure monitor and surgical technique for wireless submucosal implantation. The idea is to place a pressure-sensing element beneath the bladder mucosa to facilitate chronic bladder pressure monitoring. The device consists of a micro battery, a pressure transducer, and a custom integrated circuit with instrumentation, telemetry, and power management circuitry and was protected with PDMS gel and a conformal coating of parylene-C as a moisture barrier. The wireless pressure recordings against recordings from a catheter showed strong agreement at bladder values below 100 mL. For readings over 100 mL, values of the submucosal device diverged significantly from the reference catheter. Mucosal erosion occurred 2–4 weeks after implantation, leading to device migration into the bladder lumen and expulsion during urination. Further investigation into device miniaturisation, anchoring methods, and understanding of submucosal pressure biomechanics may enable chronic submucosal pressure monitoring.

##### Capacitance-Based Sensors

**Figure 8:**
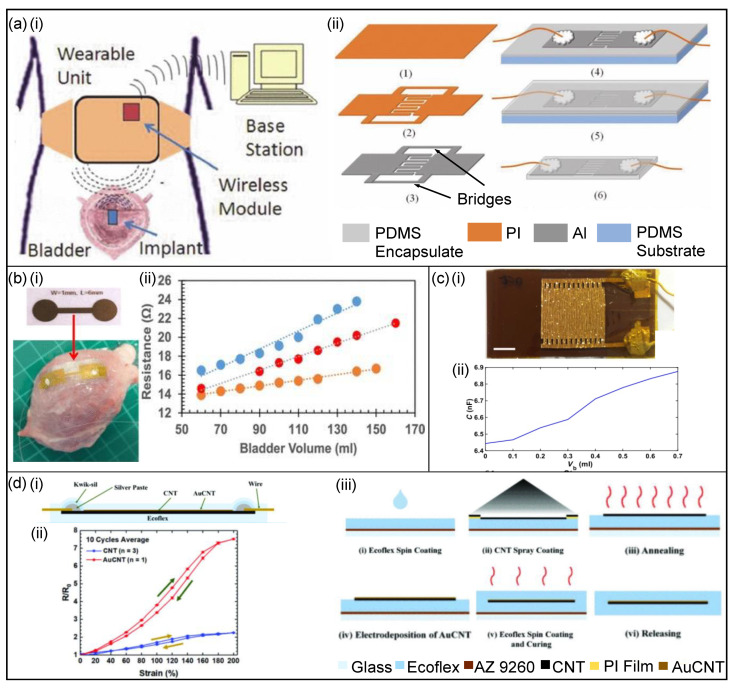
Bladder-monitoring devices. (**a**,**i**) The conceptual design of the system consisting of the capacitance-based sensor implant, an external wearable unit, and a receiver base station for data acquisition, and (**ii**) the fabrication process of the sensor [60]. Copyright 2013, IEEE. (**b**,**i**) The capacitance-based sensor, showing the top view of the wrinkled electrodes, and (**ii**) a capacitance–volume curve for bladder filling [48]. (**c**,**i**) The laser-patterned stretchable strain sensor, and (**ii**) the capacitance versus bladder volume for various sensor placements on the bladder [63]. (**d**,**i**) Cross-sectional image of the AuCNT strain sensor, (**ii**) comparative results of the CNT strain sensor versus the AuCNT strain sensor, and (**iii**) fabrication process of the strain sensor [64]. Copyright 2021, IEEE.

Cao et al. [60] have created a wireless sensor system to monitor bladder volume in small animals which can be seen in Figure 8a. The system consists of a transducer implant for signal transduction, an external wearable unit to record and relay signals, and a receiver base station for data acquisition. A passive telemetry platform was developed to employ the capacitance-based sensor in vivo in which the implant received the electromagnetic energy from the wearable unit, supplied power to operate the sensor and transduced the sensor data back to the reader in the wearable unit. Using interdigitated capacitance-based (IDC) structures can reduce the size of the sensor. As shown in Figure 8a, the IDC structures were fabricated with a 125 µm-thick polyimide (PI) film and two parts of the IDCs were held by bridges. After sputtering 4 µm-thick aluminium (Al) onto the IDC structure cut-outs, the IDCs are then attached to a PDMS layer, then wires are connected using silver epoxy. Finally, the bridges will be cut off, followed by casting a top layer of PDMS to package the entire sensor. The system can detect small changes in volume in a rat’s bladder and records data remotely via wireless communication.

Hassani et al. [48] incorporated a bladder-inspired wrinkled interdigitated capacitive sensor in a sensing-actuation system to monitor the bladder, shown in Figure 8b. The polymeric capacitive sensor consisting of wrinkled interdigitated electrodes was designed so it responded to both perpendicular and bending forces. The interdigitated gold electrodes were fabricated separately before being laminated on a 1 mm-thick double-sided adhesive acrylic pre-stretched elastomer layer. Subsequently, the elastomer layer was released and adhered to a polyimide (PI) substrate. The capacitance of the sensor would change during bladder filling and can be used as an indicator for bladder volume, shown in the capacitance versus bladder volume characteristics in Figure 8b.

##### Strain Sensors

Hannah et al. [63] produced a soft, biocompatible sensor using stretchable electronics to monitor bladder stretch. The sensor (Figure 8c) consists of a chromium-coated gold layer on a stretchable and compatible hydrogel to estimate the strain. As the bladder alters in size due to filling or voiding, the sensor stretches, causing the resistance to change—allowing for an estimation of the bladder volume. The resistance versus bladder volume characteristics are shown in Figure 8c. Additionally, the biocompatibility of the sensors was tested in vitro using pig bladder.

Another strain sensor developed by Jo et al. [64] uses Ecoflex-carbon-nanotube (CNT) and formed gold-CNT (AuCNT) fused on a CNT film to monitor bladder volume. The sensor (Figure 8d) uses Ecoflex 00-50 as the substrate material, known for its low Young’s modulus and biocompatibility, making it an appropriate material for the highly stretchable bladder. CNT is used for a resistive-type strain sensor and the AuCNT composite was used to increase the sensor’s sensitivity, with results showing a gauge factor of approximately 5-fold higher for AuCNT. The comparative results for the two sensors are shown in Figure 8d. The fabrication process is also shown in Figure 8d—a spray method was used to form the CNT film and an electrodeposition method was used for the formation of the AuCNT composite. Initially, an AZ 9260 photoresist sacrificial layer was spin-coated on a glass substrate before the Ecoflex 00-50 spin-coated on it. Subsequently, CNT film was spray-coated on the Ecoflex layer with a polyimide mask patterned by laser cutting. Wires were then bonded on the exposed CNT and sealed using Kwik-sil to prevent breakage. After the AuCNT composites were deposited using an electrodeposition method, the sensor was coated with a layer of Ecoflex and then released by dissolving the sacrificial layer. Furthermore, using Arduino, a system was developed to monitor the status of the sensor that would flash a certain colour depending on the strain of the system.

#### 3.3.2. Stimulation Devices

##### Nerve Stimulation

**Figure 9:**
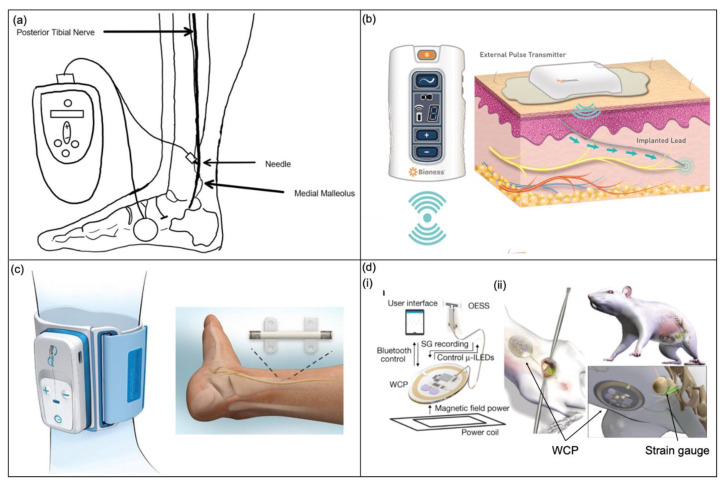
Stimulation devices to treat OAB. (**a**) Posterior tibial nerve stimulation (PTNS) device [46]. (**b**) The implanted Bio Stimrouter system with a patient programmer to control external pulse transmitter [53]. Copyright 2017, Springer Nature. (**c**) The BlueWind RENOVA system implanted adjacent to the PTN and external control unit around the ankle [53]. Copyright 2017, Springer Nature. (**d**) (**i**) A schematic of the optogenetic stimulation system—an optoelectronic stimulation and sensing (OESS) module, a low-modulus, stretchable strain gauge to monitor bladder fullness, integrated micro inorganic light-emitting diodes (μ-ILEDs) to provide optogenetic stimulation, the wireless control and power (WCP) module to record the strain gauge and controls the μ-ILEDs; (**ii**) the placement of the strain gauge around the bladder and the WCP module [43]. Copyright 2017, Springer Nature.

After failing pharmacological and physiotherapy treatments for OAB, sacral nerve stimulation (SNS) via the percutaneous tibial nerve (PTN) is a suitable next-in-line management option [46]. PTNS is a non-surgical treatment option providing a low-risk alternative to contemporary treatments for OAB. A needle electrode with a connected stimulator device sends mild electrical impulses to trigger the tibial nerve (Figure 9a) [46]. The impulses then travel to the sacral nerves responsible for bladder function; stimulating these nerves via electrical impulses can eventually lead to the modification of detrusor overactivity, usually over a series of 12-weekly treatment sessions [96]. The inaccuracy in nerve stimulation can cause pain and has limited its use.

Furthermore, there are other tibial nerve stimulation (TNS) devices being studied to treat OAB. The Bio Stimrouter system is one such device [53] and has demonstrated negligible adverse effects in patients with chronic pain [97]. The device, shown in Figure 9b, consists of an implanted lead with an integrated receiver, anchor, and three-electrode contacts in the proximity of the PTN. A rechargeable external pulse transmitter and electrode patch deliver stimulation energy to the lead transdermally. Moreover, the patient can control the device using a program that controls the external pulse transmitter with wireless radiofrequency. Another TNS device developed by BlueWind Medical is a miniature implant called RENOVA [53], shown in Figure 9c. The device can be surgically implanted or positioned under ultrasound using a designated delivery system and is battery-less and wirelessly powered by an external control unit worn on the ankle. The control unit is individually set for each patient and controls the stimulation frequencies. A study assessing the performance of the RENOVA system showed that after six months, 71% of subjects experienced 50% improvement in symptoms of OAB [98].

##### Optogenetic Stimulation

The field of bioelectronic medicine endeavours to develop engineering systems designed to relieve clinical conditions by stimulating the peripheral nervous system [99]. Mickle et al. describe a wireless closed-loop optogenetic system to stimulate the sacral nerve to treat patients with OAB [43]. Contemporary stimulation protocols, such as direct electrical stimulation, can cause pain, injury and inflammation to nerves and have been shown to target large nerve bundles, resulting in a lack of organ specificity [100,101], whereas the system proposed in the study is a bio-optoelectronic implant that avoids these issues by: (1) incorporating a precise, soft biophysical sensor for the continual measurement of organ function. (2) Using an optical stimulation interface employing microscale inorganic LEDs (μ-ILEDs) to activate and control inhibitory opsins for optogenetic neuromodulation. (3) Eliminating chronic behaviours of the body by using data algorithms and a control module. This fully implantable device, shown in Figure 9d, controls inhibitory opsins expressed in bladder sensory afferent nerve fibres with μ-ILEDs and has been shown as an effective approach to normalise bladder dysfunction in preliminary tests on mice and rats. The whole system consists of: (1) an optoelectronic sensing module incorporating a layer of silicone doped with carbon black as a strain gauge wrapped around the bladder to monitor bladder volume; (2) a pair of μ-ILEDs for optogenetic stimulation; (3) a wireless control and power (WPC) module that controls the system using a portable base station for wireless communication; (4) a wireless harvesting unit to distribute power to the whole system. Furthermore, there is specific software on the user interface that allows for real-time visualisation and provides an extensive analysis of the collected data. Finally, the entire device is encapsulated in a low-modulus silicone material to provide insulation. This technology could be furthered by utilising excitatory opsins to stimulate the bladder to alleviate people with UAB; however, more precise opsin targeting would be required to avoid causing pain by stimulating nociceptive nerves.

## 4. Underactive Bladder

### 4.1. Diagnosis

UAB or detrusor underactivity is defined as a reduction in strength and/or duration of the detrusor muscle, resulting in prolonged bladder emptying or a complete failure to achieve bladder voiding within a typical periods [102,103,104]. Estimations of the prevalence of UAB is between 9 and 23% in men under the age of 50, rising to nearly 50% in men older than 70, whereas the prevalence is approximately 12–45% in older women [105]. The conventional method for defining UAB is the postvoid residual urine volume. At present, there is no normalised quantity of postvoid residual needed for an exact diagnosis; but, overall, researchers tend to use a postvoid residual volume greater than 300 mL to diagnose UAB [106,107]; others have used 100, 400 and 500 mL [108]. The bladder can undergo large volume changes during its storage and urination phases—for storage of 500 mL, the bladder can expand up to 5-fold its emptying state volume [109]. Therefore, assistive devices aiding in bladder voiding require precise compatibility accounting for these large volume changes.

The causes and definitions of UAB are summarised in Table 4. Overall, UAB can be seen in many myogenic failures or neurological conditions—it is defined as a reduction in the strength of contraction or duration, resulting in prolonged bladder emptying or altogether failure of bladder emptying [30]. UAB is often observed when one of several mechanisms are damaged: bladder peripheral afferent and/or efferent pathways, lumbosacral spinal cord, and myogenic failure [110]. One leading cause of UAB is due to spinal cord injuries (SCI). The prevalence of SCIs on a global scale is between 236 and 1009 per 1,000,000 people [111], and up to 80% of patients with SCI suffer from various grades of bladder dysfunction [24] and require various assistive treatments to void the bladder.

### 4.2. Clinically Approved Management Strategies

#### 4.2.1. Conservative Management Strategies

Like OAB, many of the causes of UAB are unforeseeable, suggesting there is no exact way to prevent UAB. However, there are some treatment options available, although none is an absolute cure—they only act to slow down the disease and help limit the damage to the bladder and kidneys. Presented in Table 5 are the current treatment options to manage UAB. The most common treatment for UAB is conservative management—this involves intermittent and indwelling urinary catheters for willing and physically able patients or medicine [24]. An indwelling catheter is inserted into the bladder via the urethra and remains in place continuously, whereas an intermittent catheter is inserted each time the user needs to void. Intermittent catheterisation may induce a greater increased risk of damage to the urethra due to unsuccessful placement [112]. Zhang et al. conducted a review of intermittent versus indwelling urinary catheterisation and concluded that there is no increase in the risk of contracting a UTI [113]. However, an intermittent catheter is less debilitating and provides the patient with more autonomy and fewer barriers for intimacy, thus improving quality of life [114]. Furthermore, catheterisation several times a day can severely impact patients’ mental health. Quality of life was studied in patients with spinal cord injuries—using an indwelling urinary catheter or performing intermittent catheterisation was associated with the worst scores for mental health [115].

In contrast, the user may opt for treatment with medicine. Standard pharmacotherapy includes the use of α-adrenergic blockers and muscarinic agonists (bethanechol), which act to reduce urethral outlet resistance [23]. Anticholinergic medications are usually the first line of therapy and can occasionally show promising results. They work by blocking cholinergic transmission at muscarinic receptors. However, the side effects are severe, for example impairment of memory and cognition, tachycardia and arrhythmias caused by prolonged QT intervals (i.e., the time from the start of the Q wave to the end of the T wave on the ECG), visual blurring, xerostomia, and constipation [24].

Due to the pharmacological side-effects and the somewhat traumatic experience of intermittent and indwelling urinary catheters, alternative methods to assist in urinary voiding are being explored. Assistive devices are being explored as a potential new option to replace current physical and pharmacotherapy treatments, providing a more manageable and less physically and mentally debilitating treatment, enabling the user to live a standard quality of life.

#### 4.2.2. Inflow™ Intraurethral Valve Pump

**Figure 10:**
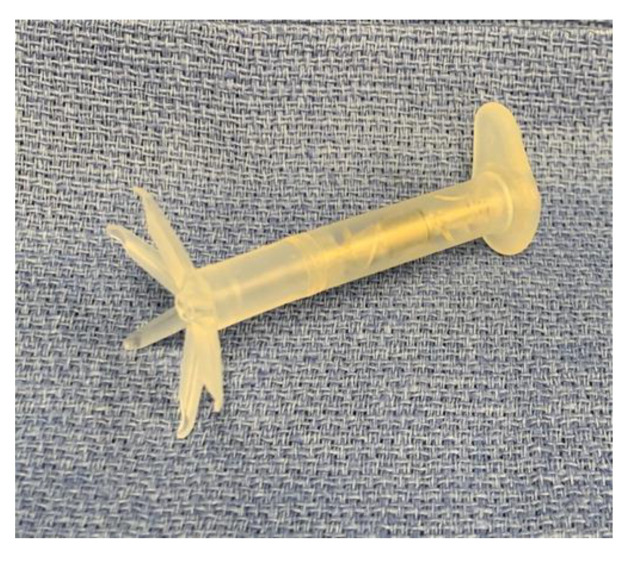
The inFlow™ intraurethral valve pump for women [39]. Copyright 2020, Taylor and Francis.

Figure 10 shows the inFlow™ intraurethral valve pump and activator [39]—it has been designed for women with impaired detrusor contractility. The inFlow is comprised of a short self-retaining silicone catheter containing an internal valve and pump mechanism which uses a miniature magnetically-coupled pump activated by a handheld remote control. The device is inserted into the female urethra and works to empty the bladder; the patient holds the remote control over the pelvis and activates it by pushing the button. The remote activates a miniature magnetically coupled pump inside the device which in turn pumps urine from the bladder. Upon void completion, the button is released, and a valve is activated that blocks the additional flow of urine. After a month, the device is to be removed and replaced with a new one. This device provides novel technology to assist in bladder voiding, although the inFlow does require manual dexterity of the user for the placement and control of the device.

### 4.3. Research-Based Solutions

#### 4.3.1. Stimulation Devices

Various stimulation devices can be utilised to induce bladder voiding. These stimulation devices can be subdivided into: (1) nerve stimulation involving the stimulation of the sacral nerve and the percutaneous tibial nerve. (2) Electrical muscle stimulation that triggers the bladder muscle directly to induce voiding. (3) Optogenetic stimulation includes a biological technique that involves the use of light to control neurons that have been genetically modified.

##### Nerve Stimulation

**Figure 11:**
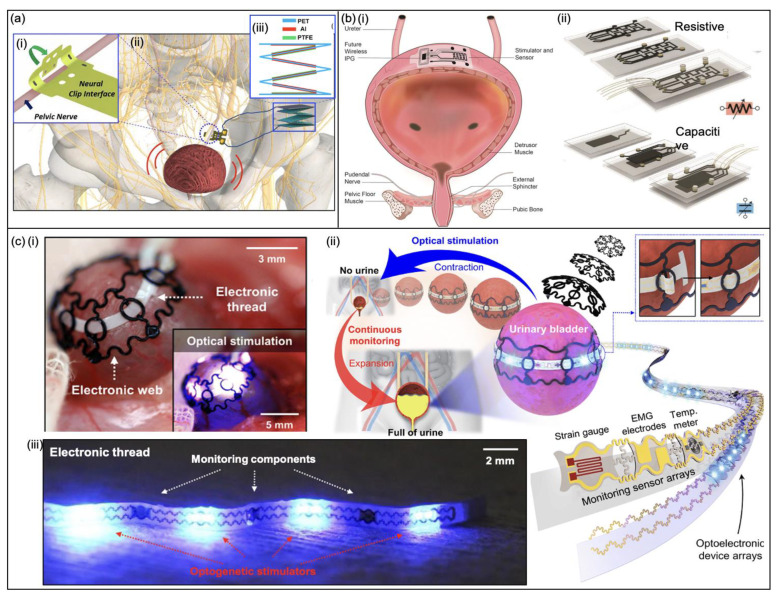
Stimulation devices for UAB. (**a**,**i**) The FNC neural chip implanted on the peripheral nerve, (**ii**) the mechano-neuromodulation pelvic nerve stimulation system using a triboelectric neurostimulator, and (**iii**) the four-layer stack triboelectric nanogenerator (TENG) [65]. Copyright 2019, Nano Energy. (**b**,**i**) Ultracompliant carbon-nanotube direct bladder device schematic, and (**ii**) fabrication process of the resistive-type sensor and capacitive-type sensor [66]. Copyright 2019, John Wiley and Sons. (**c**,**i**) Optogenetic stimulation device integrated onto the bladder in a rodent, (**ii**) the electronic and optoelectronic components forming the E-thread, and (**iii**) an image of the E-thread emitting blue light [44]. Copyright 2020, the authors. Reprinted with permission from AAAS.

In one study, Lee et al. [65] demonstrated mechano-neuromodulation of autonomic pelvic nerves using a triboelectric neurostimulator integrated with a flexible neural chip interface. They use the concept that mechanical movements in the body can be used as an energy source for neuromodulation by combining a stacked triboelectric nanogenerator (TENG) and flexible neural chip (FNC) interface for bladder modulation. They demonstrate modulation of bladder function by implanting and clipping an FNC interface onto the pelvic nerve, shown in Figure 11a. The fabrication process of the FNC followed standard photolithographic procedures and consisted of a polyimide–gold–polyimide sandwich structure—allowing for reliable implantation onto small pelvic nerves (Figure 11a). The gold electrodes were then coated with iridium oxide to increase stimulation performance. Furthermore, the TENG had a zig-zag shape and was fabricated using polyethylene terephthalate (PET) as the body and aluminium-polytetrafluoroethylene (Al-PTFE) as the contact triboelectric pair. A total of four layers were prepared to maximise output current for effective stimulation. Different frequencies of hand-tapping were applied to the TENG within the range of 25 to 150 BPM (beats per minute) to produce nerve stimulation signals. During in vivo experiments, stimulation signals of above 50 BPM were sufficient to void the bladder. Long-term implantation of this device may lead to performance degradation due to foreign-body reactions and scar tissue.

##### Electrical Muscle Stimulation

Figure 11b presents the schematic of a bladder control concept consisting of sensors and stimulation electrodes [66]. Yan et al. demonstrated two types of organ strain-sensing devices (Figure 11b)—one is a piezoresistive sensor and the other is a capacitive strain sensor. The piezoresistive device consists of one CNT layer and the capacitive device has two CNT layers. Additionally, each sensor had four platinum–silicon composite electrodes to induce bladder contractions and both devices were insulated in silicone (Ecoflex) for durability. The resistance and capacitance values of the sensors increase with increasing strain as the bladder fills. The resistive-based sensor was chosen as a more robust and easier sensor to implement for in vivo experiments, mainly due to the strong correlation of resistance versus bladder volume and ease of sensor calibration. With a stimulating drive voltage of approximately 10 V and eight bladder emptying cycles per day, a commercial 350 mAh lithium battery (EaglePicher LTC-3NP-M1) could potentially power the device for over seven years without recharging.

##### Optogenetic Stimulation

Jang et al. [44] created a system that integrates onto the urinary bladder without adhesive to enable precise monitoring and optogenetic manipulation to assist with UAB. The expandable device can be seen in Figure 11c. The system is a combination of two components forming an E-thread: a web-type elastomeric framework that envelopes the entire bladder and maintains seamless contact during bladder filling and emptying, and a narrow thin electronic strip that includes μ-LEDs to induce contractions. To induce muscle contraction, adeno-associated virus (AAV) encoding ChR2 tagged with an enhanced green fluorescent protein (eGFP) [AAV9-CAG-hChR2(H134R)-eGFP] is injected into a region between detrusor muscle layers. Subsequently, blue light was emitted, which induced muscle contraction, shown in Figure 11c.

#### 4.3.2. Prosthetic Devices

Another method to assist with urological conditions affecting the urinary system is bladder actuation systems. The proceeding sections will delve into assistive actuation devices to help void the bladder in people who suffer from UAB.

##### Shape Memory Alloy-Based Bladder Actuators

**Figure 12:**
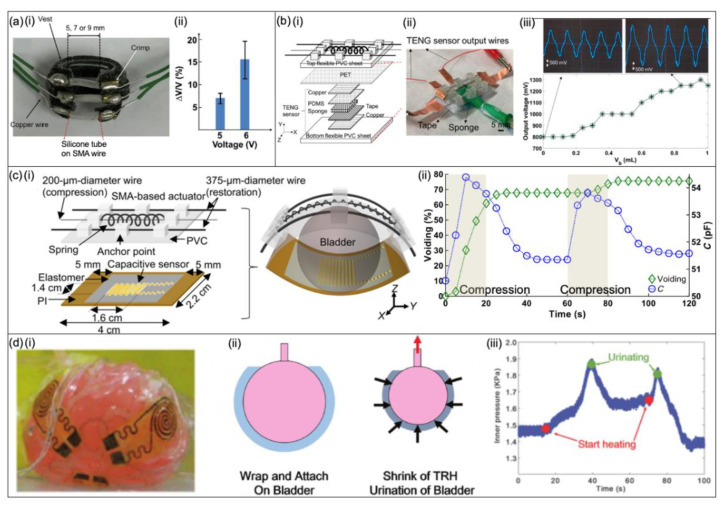
Bladder actuation devices. (**a**,**i**) Schematic of the 3D printed SMA-based actuation device, and (**ii**) the voiding percentages for 5 and 6 V activations of the device [47]. (**b**,**i**) A schematic view of the actuation device, (**ii**) an image of the device on a rubber balloon, and (**iii**) output voltage versus balloon volume characteristics of the TENG sensor [116]. (**c**,**i**) The dimensions of the device and capacitive sensor with a schematic of the device around the bladder, and (**ii**) the voiding and capacitance characteristics of the integrated bladder system during the compression phase [48]. (**d**) (**i**) The assistive detrusor device, (**ii**) the operation process of the device, and (**iii**) a graph showing the pressure in the bladder during the shrinking and relaxing phases of the device [52]. Copyright 2018, John Wiley and Sons.

One implantable 3D printed device that incorporates shape memory alloy (SMA)-based actuators was shown to have a voiding capability of more than 8% of the bladder (Figure 12a) [47]. The target demographic for this device is people with myogenic UAB—a condition where the patients have degraded detrusor muscle function, resulting in an inability to initiate voiding the bladder. The device is comprised of three SMA wires with brass crimps designed to be placed around the bladder to apply a force, resulting in bladder voiding. The SMA wires are covered in silicone tubing to provide thermal insulation to reduce heat transfer to the bladder. This device was the first flexible 3D printed medical device driven by SMA actuators, but it only provided a voiding volume of 8% in one activation cycle, shown in Figure 12a. In the next version of this SMA-based actuator, the voiding efficiency was improved to 25% [49], but achieving a larger voiding efficiency required a new bi-stable mechanism with the help of an SMA spring [116]. The spring actuator was also integrated with a flexible TENG sensor to detect the fullness of the bladder as shown in Figure 12b. The actuator consists of two flexible PVC sheets for the top and bottom with a layer of PET in between. The TENG sensor is a sandwich design between the PET and the bottom PVC layer. The TENG sensor consists of top and bottom copper electrodes and layers of PDMS and a 1 mm-thick sponge containing water in between. The bending of the TENG sensor due to bladder filling squeezes the sponge so more water is in contact with the PDMS layer, thus increasing the output voltage. Output voltage versus volume characteristics of the TENG sensor are shown in Figure 12b.

The most recent device by Hassani et al. [48] achieved a high voiding rate of 70–100% of a rat’s bladder (Figure 12c). The paper shows an advancement in technology by amalgamating a soft and thin capacitive sensor with the SMA-based actuator to achieve a closed-loop control for the bladder. As the bladder volume increases, the capacitance increases and the capacitance change can be used to estimate the fullness of the bladder, as explained previously in Figure 8c. The device consists of a flexible SMA-based actuator and a capacitive interdigitated sensor. The sensor’s 125 μm-thick PI substrate is connected by using 70 mm-thick Kapton tape for the width of both side edges to the actuator’s 180 μm-thick PVC substrate. PVC substrate contains the SMA spring component used to compress and void the bladder, and SMA wires to return the PVC substrate to a flat shape after voiding. The capacitive sensor is positioned on the inner surface of the PI substrate and is kept in contact with the bladder surface by the aid of the embedded spring, mitigating the need for direct adherence. The voiding and capacitance characteristics during the voiding phase are displayed in Figure 12c and show a voiding percentage greater than 70%.

##### Hydrogel-Based Devices

Yang et al. [52] presented an actuation device consisting of a composite hydrogel membrane that wraps around the bladder to mimic the detrusor (Figure 12d). The composite hydrogel is comprised of a non-responsive tough hydrogel (NRTH), a thermoresponsive hydrogel (TRH) and silk scaffolding to envelop the bladder, to actuate, and to squeeze a sphere-shaped bladder when heated. The properties of hydrogel can be fine-tuned by the selection of material, allowing them to swell or to shrink in response to an external stimulus [117]—in this instance, temperature. Flexible Joule heaters made of thin copper conductive film coated with PDMS and PI polymers were integrated within the composite hydrogel. A 12 V voltage was applied to the Joule heaters to heat the TRH for shrinking and voiding of the bladder. The shrinkage of the hydrogel changed the resistance of the copper film that was then transferred via an Arduino chip to a mobile app for estimating the bladder volume. The changes in the inner pressure of the bladder during heating of the TRH are also presented in Figure 12d.

## 5. Other-Urinary-Affecting-Disorders

### 5.1. Diagnosis

The following sections present other-urinary-affecting disorders—these are urological disorders that do not fall into the category of OAB or UAB, but can still disrupt the normal function of the urinary system if left untreated and need novel assistive electronic solutions.

#### 5.1.1. Kidney and Ureteral Stones

Kidney stone disease is a crystal concretion commonly formed within the kidneys and is the most common disease of the urinary tract [118]. Kidney stones become ureteral stones when moved from the kidney into the ureter and disrupt the flow, which can generate a considerable amount of pain. Moreover, when the stone gets close to the bladder, there may be a constant need to pass urine and can also cause burning when voiding, pain at the tip of the urethra and blood in the urine. Kidney stones have been linked to an increased risk of chronic kidney disease, renal failure, cardiovascular disease, diabetes and hypertension [119,120,121,122]. Kidney stones affect approximately 12% of the world population at some stage in their lifetime—and can affect all people but occur more often in men than women between the ages 20 and 49 years [123,124]. Most small stones that cause minimal symptoms do not require invasive treatment and can usually be managed by drinking water to dilute urine, taking pain relievers to assist the passing of small stones and medical therapy such as alpha-blockers to relax the ureter to help pass the stone [125]. If the stones persist, extracorporeal shockwave lithotripsy (ESWL) can be used. ESWL involves firing shockwaves through the skin and focusing them to break the stones into smaller fragments that can pass naturally; this involves either ultrasound or x-ray [126]. If ESWL is unlikely to fragment the stones further, other surgical methods should be considered.

#### 5.1.2. Cancer

There are several types of urological cancers, including bladder cancer and renal cancer in both men and women and prostate cancer, testicular cancer, and penile cancer in males. Furthermore, prostate cancer is the most common cancer in men and the second leading cause of death in males [127].

Frequently, symptoms do not occur until cancer has become more advanced—blood in urine is a common symptom for bladder, kidney, and prostate cancer and many patients with prostate cancer have altered urination and sexual function. Treatment for urological cancer depends on many factors such as tumour grade and stage. Common treatment options for urological cancers are: surgery, radiation therapy, hormonal therapy and chemotherapy [10]—all have their own benefits and shortcomings. A treatment strategy integrating many of these management systems may be beneficial; however, the selection of a strategy can depend on many factors, such as patient preferences and quality of life. Robotic assistive surgery may be an option for patients who require surgical intervention [10].

#### 5.1.3. Benign Prostatic Hyperplasia

BHP is the leading cause of acute urinary retention (AUR) [128]. Autopsy studies have seen a histological prevalence of 8% for men in their 30s, increasing to 50% and 80% for men in their 50s and 80s, respectively [4]. The initial management of BPH usually consists of a supra-pubic or urethral or urinary catheter, proceeded by hospital discharge and patient follow-up [129]. If symptoms persist further, action is required, and this usually involves pharmacotherapy—a course of drugs to relax the smooth muscles found in the bladder [129,130]. Surgical prevention can be considered for patients with persistent AUR due to BHP if required [129].

#### 5.1.4. Interstitial Cystitis

Interstitial cystitis (IC) is a condition in which patients experience debilitating chronic pain in the pelvic area, which can include pain during bladder filling and causes increased urinary urgency [130]. Currently, IC is a syndrome of unknown aetiology but can have a profound negative impact on quality of life [131]. In the past, IC was diagnosed infrequently with a global prevalence of approximately 0.1% [132]; however, the prevalence has increased to approximately 2% among females due to broader diagnosis criteria [133]. Traditionally, there has been a deficiency of expertise to study this syndrome and numerous treatment options fail to cure or change the natural history of the disease [134]. The recent development in optogenetic stimulation may provide a future for the management of IC.

### 5.2. Clinically Approved Management Strategies

#### 5.2.1. Robotic Surgery

For many urological diseases, surgery remains a safe and reliable option. In the last decade, there has been development in minimally invasive surgery through the introduction of robot-assisted surgery [135,136]. This section will present robotic surgery techniques used for the management of urological conditions.

#### 5.2.2. Robot-Assisted Ureteroscopy

**Figure 13:**
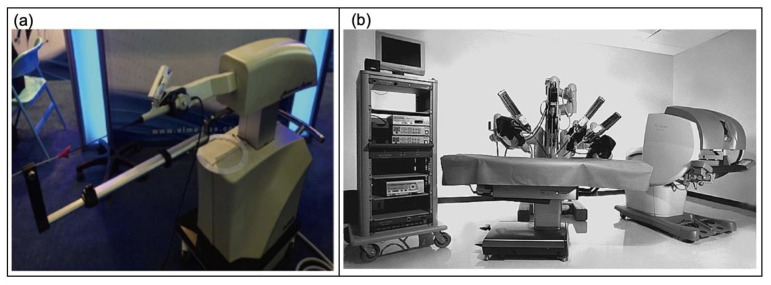
Robotic surgery instruments. (**a**) The robotic flexible ureteroscopy machine [54]. (**b**) The da Vinci surgical system. From left to right: image processing stack, a patient-side cart containing robotic arms and the surgeon’s console [138]. Copyright 2007, IEEE.

Since the 1990s, ureteroscopy (URS—a procedure in which a small scope is inserted into the bladder and ureter) has become common for stone removal. With the advancement of technology, there has been an introduction of robotic flexible ureteroscopy [54]. This method allows the surgeon to command their flexible ureteroscopy and laser fibre via a robotic console shown in Figure 13a. The surgeon uses a console and a joystick to control the URS—the major benefit of using robot flexible ureteroscopy seems to favour surgeon ergonomics. Saglam et al. [137] presented the Roboflex Avicenna as a safe system for robotic flexible ureteroscopy and out of 81 patients, only 1 required further treatment due to a malfunction of the system.

#### 5.2.3. Robot-Assisted Prostatectomy

The most regular procedure using robotic surgery is radical prostatectomy, where the da Vinci surgical system has been the benchmark for robotic procedures [55]. The system has three components: the surgeon console, a patient-side cart containing robotic arms and an image processing or insufflation stack as shown in Figure 13b.

Menon et al. [139] reported that 95% of 1100 patients under surgery by the system were discharged within the first 24 h, with an operative time of only 70–160 min and blood loss of 50–150 mL, which is considerably less than patients having similar treatments using laparoscopic techniques.

### 5.3. Research-Based Solutions

#### 5.3.1. Prosthetic Devices

In some extreme situations, there is a need to replace part of the urinary system entirely—this section will explore assistive electronic devices to replace the sphincter and the bladder.

##### Artificial Urinary Sphincter

Since the 1970s, the artificial urinary sphincter (AUS) has remained the gold standard for the treatment of many cases of urge urinary incontinence (UUI) due to benign prostatic enlargement [140]. AUS is also a treatment option for people with other urological conditions such as OAB.

**Figure 14:**
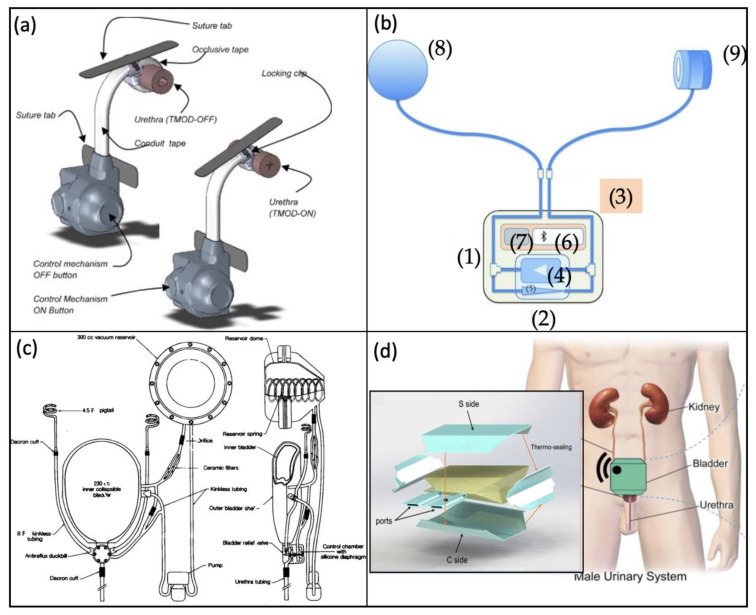
Prosthetic devices. (**a**) Tape mechanical occlusive device (TMOD) [67]. Copyright 2011, Elsevier. (**b**) Remote-controlled hydromechanical artificial urinary sphincter: (1) pumping system, (2) hydraulic unit, (3) control unit, (4) piezoelectric pump, (5) latched solenoid microvalve, (6) Bluetooth 2.1 communicating microcontroller, (7) lithium battery, (8) pressure-regulating balloon, and (9) urethral cuff [68]. (**c**) Artificial bladder concept—diagram of the anterior and lateral view [50]. Copyright 1992, Elsevier. (**d**) A schematic of the fully implantable smart artificial bladder [51]. Copyright 2021, IEEE.

Malaeb et al. [67] have developed a tape mechanical occlusive device (TMOD)—it uses a spring-loaded mechanism to apply constrictive circumferential pressure on the urethra (Figure 14a). The device consists of a titanium casing housing a nickel–cobalt–chromium alloy spring that is biocompatible, and applies tension to Teflon-coated polyester sutures running through the conduit and occlusive tapes. When the device is ON, the occlusive tape constricts and applies radial pressure to the urethra; the user would then press the OFF button to remove the occlusive pressure from the urethra to allow for voiding.

There have been developments of remote-control activation mechanisms for AUS. Biardeau et al. [68] proposed a novel remote-controlled hydromechanical AUS. The device has been designed to replace the manual pump with a remotely controlled pump system (Figure 14b). The hydraulic module consists of a piezoelectric micropump and a latched solenoid microvalve mounted in parallel. The control unit has a Bluetooth 2.1 microcontroller for wireless communication, as well as a rechargeable lithium battery—with an estimated seven micturitions per day up to 41 days. Finally, all the components are mounted in a silicon-coated acrylonitrile butadiene styrene (ABS) case and replace a manual pump. This system can be mounted in parallel with other similar AUS to maximise performance. The AUS has four distinctive states—closed state, cuff deflation, open state, and cuff inflation. When the device is in its closed state, the cuff is inflated to provide constant pressure to the urethra, and the pump is unpowered. When there is a Bluetooth signal connected, the pump is activated to move fluid from the cuff to the balloon—the cuff is now deflated, and the system is in its open state allowing micturition. Finally, the cuff is then inflated to provide pressure to the urethra once again.

##### Artificial Bladder

In extreme situations, the bladder may have to be fully removed, for example in the case of radical cystectomy due to cancer of the bladder. In this unfortunate circumstance, the standard treatment is either urinary system diversion, or to replace the organ with autologous tissue from the intestine [141]. This form of treatment can have severe complications such as the risk of developing carcinomas and has high rates of re-operation, leaving the patient physically and mentally impaired.

The concept of artificial bladder replacement was proposed in the 1990s—Barrett et al. [50] proposed a new design concept presented in Figure 14c. The components are: a ureter made of silicone tubing reinforced with a nylon spiral which prevents kinking—its proximal end is inserted into the renal pelvis, and at its distal end the ureter is attached to a polysulfone housing unit that attaches to a urethral valve unit. The bladder is constructed of two shells—a 230 mL flexible silicone inner bladder and a 300 mL rigid polysulfone outer shell. The reservoir has an 11 cm stainless steel spring inside the reservoir creating negative pressure within the entire system when compressed. The flow resistors regulate the rate of refilling of the reservoirs. The urethral valve is divided into two main chambers: one is connected to both urethra and the bladder and acts as the conduit for the urine, and the other is connected to the reservoir and the pump to open and close the valve.

A more recent paper proposes a fully implantable smart artificial bladder system that collects urinary fluids and provides the patient with real-time feedback (Figure 14d) [51]. The system is designed to be stretchable, enabling it to be treated with urine-resistant coating and has built-in passive check valves preventing back-flow to the kidneys. It is comprised of four flexible yet unstretchable multilayer planar walls, with the lateral walls designed to self-fold like a pre-shaped bag. The device is provided with four electromagnetic distance-sensing units and a control unit to estimate the amount of fluid collected. The device is placed inside the patient in lieu of the natural bladder and is surgically anchored to the pubic bone, allowing voiding through abdominal contraction, such as the Valsalva manoeuvre. By considering the unstretchability of the system, volume measurements can be based on the distances of the bag walls rather than strain or pressure. Although this device has shown promising results for a replacement bladder, showing a relative error of 15% when testing the robustness of volume reconstruction, it is battery powered and can only guarantee a lifespan of 1.5–10 years, meaning the patient would require future operations.

#### 5.3.2. Stimulation Devices

##### Optogenetic Stimulation

**Figure 15:**
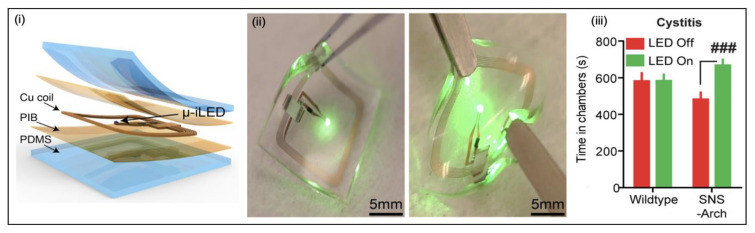
Optoelectrical system for interstitial cystitis. (**i**) Schematic depiction of the layered optoelectronic device, (**ii**) demonstrating the flexibility of the device using forceps, and (**iii**) a graph showing that SNS-Arch mice display a significant response for LED ON compared to LED OFF, whereas wild-type mice have no preference (### *p* = 0.0065, 11 mice per group, two-way analysis of variance (ANOVA)) [45].

Samineni et al. [45] developed a fully implantable, flexible and wirelessly powered optoelectronic stimulation system for the optogenetic inhibition of nociceptive sensory afferents to alleviate pain caused by IC. The activation of archaerhodopsin (Arch—a light-activated proton pump) by green light is known to suppress neuronal firing by inducing membrane hyperpolarisation [142]. The device (Figure 15) is implanted subcutaneously between the skin and bladder muscle and powered by near field communication (NFC) and is comprised of several layers. An 18 µm-thick copper foil, μ-LEDs and mounted chips are encapsulated in a bilayer of polyisobutylene (PIB) and PDMS. The layer of PIB provides protection against moisture and enables the device to operate for at least four weeks after implantation, with many of the devices remaining functional for six months or more. Furthermore, the electric system includes a rectangular coil with surface mounted chips to enable power transfer via magnetic control to a loop antenna. During experiments, the light activation of Arch under control of the sensory neuron specific sodium channel (SNS) gene showed a significant response (Figure 15). This technology would need additional modification to meet requirements for use in clinical settings. However, it suggests that optogenetic manipulation of nociceptive bladder afferents can alleviate pain associated with IC.

## 6. Conclusions

Technological development in flexible electronics has helped develop new solutions for existing urological conditions. Many of these devices intend to replace contemporary conservative management strategies for OAB, UAB and OUAD. Common conservative management strategies such as catheterisation and pharmacotherapy often leave patients mentally and physically impaired and fail to provide a satisfactory standard of living.

Currently, there are a few distinctive areas in which the field has advanced. (1) Robot-assistive surgery is becoming commonplace for ureteroscopy and prostatectomy. This method is a more minimally invasive technique, where the surgeon uses a console and a joystick to operate, providing more accuracy and surgeon ergonomics. (2) Bioimpedance, resistive, piezoresistive, MEMS and capacitance-based sensors, and ultrasound scanner and near-infrared spectroscopy systems are being utilised to monitor bladder volume in real time without inserting catheters with pressure sensors directly into the urethra. These systems are a mixture of invasive and non-invasive measuring techniques. Non-invasive techniques such as ultrasound near-infrared spectroscopy measure bladder volume from the skin and can be a convenient solution; however, they can have relatively low precision. (3) Various stimulation devices have been developed to induce or prevent bladder voiding. These devices fall into nerve, optogenetic and electrical muscle stimulation categories. Sacral nerve stimulation can modify detrusor overactivity, whereas stimulation of the pelvic nerves can induce bladder voiding. Similarly, optogenetic stimulation can be used to treat OAB or UAB to stimulate either inhibitory or excitatory opsins to prevent bladder voiding or induce bladder voiding, respectively. Furthermore, flexible electrodes have been used to induce electrical bladder muscle stimulation for UAB. (4) Prosthetic devices have many applications in urology. Shape memory alloy-based and thermoresponsive hydrogel actuation devices are being developed for UAB. In some extreme cases of OUAD, there is a need to replace part of the urinary system in its entirety. In these cases, artificial bladders and sphincters are being explored.

In urology, there is movement away from conservative management strategies for OAB, UAB and OUAD to new technologies that incorporate soft flexible electronics and biocompatible materials. With the continuous improvement of materials and power-harvesting techniques, these devices will become standard in the near future.

## Figures and Tables

**Table 1:** Selected papers for this study from different libraries.

Digital Library	Number of Articles
PubMed	26
IEEE	9
NCBI	9
Nature	3
Other	15
Total	62

**Table 2:** Causes and definitions for OAB.

Causes	Definitions
Neurogenic dysfunction [75]	A reduction in inhibitory neural impulses and an increase in the afferent impulses from the bladder trigger voiding reflex.
Myogenic dysfunction [76]	Altered structure or disordered function of the detrusor smooth muscle by an increase in sensitivity to cholinergic stimulation can lead to increased random activity.
Autonomous bladder theory [77]	An alteration of phasic activity is generated by muscarinic stimulation.
Other [78,79]	UTI, weak sphincter, bladder abnormalities, diabetes mellitus, excessive caffeine or alcohol and hypercalcemia.

**Table 3:** Conservative management strategies for OAB.

Lifestyle [5,21]	Pharmacotherapy [5,29]	Physiotherapy [5,22]
Reduce intake of caffeine	Antimuscinaries	Pelvic floor muscle training
Alter daily fluid intake	Oxybutynin	Bladder training
Weight loss	Propiverine	Double void
Absorbent pads	Tolterodine	Vaginal weight training
Smoking cessation	Trospium	
Scheduled toileting	Solifenacin	
Bowel regime		

**Table 4:** Causes and definitions for UAB [30].

Causes	Definitions
Nerve damage	Damage to the peripheral nerves may lessen or eliminate one’s ability to feel the filling of the bladder.
Diabetes	Increased high blood sugar can cause damage to peripheral nerves, resulting in incomplete emptying of the bladder.
Pelvic surgery	Surgery may lead to damaged nerves, causing decreased bladder contractions.
Aging	The volume and elasticity of the bladder tissue can decrease by aging.
Obstruction	An enlarged prostate or prostate cancer in men and vaginal prolapse in women can block urine flow.
Urinary tract infection (UTI)	An infection present in the bladder or urethra can lead to urinary retention.
Medications	Drugs with antimuscarinic properties block a chemical that relaxes the muscle, e.g., antidepressants, antihistamines, and muscle relaxants.
Spinal cord injury (SCI)	Injuries below lumbar vertebrae (L1) may have a flaccid bladder, which will not contract.
Other	Multiple sclerosis, Parkinson’s disease, herniated disc, lesion of the pudendal nerve, acquired immunodeficiency syndrome (AIDS) and neurosyphilis.

**Table 5:** Conservative management strategies for UAB.

Physiotherapy [20,30]	Pharmacotherapy [23]	Catheter [20,30]
Double void	α-adrenoreceptor antagonist	Indwelling catheter
Strain to void	Muscarinic receptor agonist	Intermittent catheterisation
	Acetylcholinesterase	
